# Efficacy and Safety of Ticagrelor Versus Clopidogrel in Patients With Chronic Coronary Syndrome Undergoing Percutaneous Coronary Intervention: A Systematic Review and Meta-Analysis

**DOI:** 10.7759/cureus.86107

**Published:** 2025-06-16

**Authors:** Faisal Khan, Syed Ashraf Abid, Rutva Jani, Hafiza Zanish Iram, Manpreet Kaur Dhanjal, Sandipkumar S Chaudhari, Mohammed Qasim Rauf, Shamsha Hirani

**Affiliations:** 1 Medicine, Dow University of Health Sciences, Karachi, PAK; 2 Medicine, Deccan College of Medical Sciences, Hyderabad, IND; 3 Internal Medicine, C U Shah Medical College and Hospital, Gujarat, IND; 4 Internal Medicine, Services Hospital Lahore, Lahore, PAK; 5 Medicine, Adesh Institute of Medical Sciences and Research, Ludhiana, IND; 6 Cardiothoracic Surgery, University of Alabama, Birmingham, USA; 7 Family Medicine, University of North Dakota School of Medicine and Health Sciences, Fargo, USA; 8 Orthopedic Surgery, Hillingdon Hospital, London, GBR; 9 Cardiology, Baqai Hospital, Karachi, PAK

**Keywords:** chronic coronary syndrome, clopidogrel, dual antiplatelet therapy, percutaneous coronary intervention, ticagrelor

## Abstract

Dual antiplatelet therapy (DAPT) is essential post-percutaneous coronary intervention (PCI), yet the optimal P2Y₁₂ inhibitor for chronic coronary syndrome (CCS) remains debated. While ticagrelor demonstrates superior efficacy in acute coronary syndromes (ACS), its role in CCS is unclear. This systematic review and meta-analysis compared ticagrelor versus clopidogrel in CCS patients undergoing PCI. We searched PubMed, Embase, Web of Science, and Cochrane CENTRAL from inception to May 2025. Five studies (two randomized controlled trials (RCTs) and three observational) comprising 32,152 patients were included. Though ticagrelor showed lower all-cause mortality compared to clopidogrel (0.99% vs. 1.26%; RR 0.76, 95% CI: 0.57-1.01), this difference was not statistically significant. Similarly, no significant differences were observed in major adverse cardiovascular events (MACE) (6.23% vs. 6.48%; RR 0.90, 95% CI: 0.70-1.15) or myocardial infarction (MI) (2.67% vs. 1.43%; RR 1.17, 95% CI: 0.94-1.44). Revascularization rates were numerically lower with ticagrelor (3.66% vs. 4.82%; RR 0.83, 95% CI: 0.68-1.01) but not significantly. Notably, ticagrelor significantly reduced stent thrombosis (RR 0.50, 95% CI: 0.26-0.97) without statistically significant increases in minor bleeding (RR 1.66, 95% CI: 0.97-2.85) or major bleeding (RR 1.28, 95% CI: 0.87-1.89). These findings suggest ticagrelor may offer advantages in preventing stent thrombosis but without significant differences in mortality, MACE, or MI compared to clopidogrel in CCS patients. While bleeding risk appears numerically higher with ticagrelor, differences were not statistically significant. Given limited high-quality data specifically for CCS patients, individualized antiplatelet selection balancing thrombotic and bleeding risks remains crucial, with larger RCTs needed to confirm these findings.

## Introduction and background

Chronic coronary syndromes (CCS) represent a spectrum of clinical conditions characterized by stable atherosclerotic coronary artery disease, including stable angina and asymptomatic myocardial ischemia [1]. Percutaneous coronary intervention (PCI), a procedure to open narrowed coronary arteries, remains a cornerstone in the management of patients with CCS, aimed primarily at relieving angina symptoms and improving quality of life [2]. Dual antiplatelet therapy (DAPT), which combines aspirin with a P2Y₁₂ inhibitor, a drug that blocks platelet activation to prevent blood clots, is critical for preventing stent thrombosis (the blockage of a coronary stent by a clot) and adverse cardiovascular events after PCI. While clopidogrel has traditionally been used for patients with CCS, newer and more potent P2Y₁₂ inhibitors such as ticagrelor and prasugrel, offering faster onset and more consistent platelet inhibition, are preferred in ACS due to their superior efficacy [3,4]. However, their role in CCS remains contentious, with guidelines favoring clopidogrel for this population. 

The pharmacological distinction between these agents is notable. Clopidogrel, a prodrug requiring hepatic activation via CYP2C19, exhibits variable responses due to genetic polymorphisms [5]. In contrast, ticagrelor (a reversible P2Y₁₂ antagonist) and prasugrel (an irreversible inhibitor with a more predictable metabolism) provide stronger, more reliable platelet inhibition [6]. Despite these advantages, their use in CCS is not universally endorsed due to concerns about bleeding risks and uncertain benefits in lower-risk populations [3]. Recent observational studies and registries report increasing off-label use of ticagrelor in CCS, particularly in complex PCI cases, without significant differences in one-year mortality or myocardial infarction (MI) rates compared to clopidogrel [7]. Conversely, a large retrospective cohort study suggested improved cardiovascular survival with these agents [8] highlighting ongoing debate. 

Current European Society of Cardiology guidelines recommend six months of DAPT with clopidogrel for CCS post-PCI, reserving ticagrelor primarily for ACS [4]. This stratification stems from limited randomized evidence in CCS populations, as major trials like PLATO and TRITON-TIMI 38 focused on ACS [3,8]. Emerging data from real-world studies reveal critical knowledge gaps: while anatomical complexity often drives ticagrelor/prasugrel use in CCS [7,9], their net clinical benefit, balancing ischemic protection against bleeding risk, remains unquantified. Furthermore, heterogeneous study designs and endpoints across existing literature complicate comparative assessments. 

This meta-analysis addresses the absence of prior systematic evidence synthesis specific to CCS populations, conflicting findings from individual observational studies, and the lack of adequately powered randomized trials in this setting. This systematic review and meta-analysis aim to synthesize contemporary evidence comparing ticagrelor versus clopidogrel in CCS patients undergoing PCI. By evaluating efficacy endpoints and safety outcomes, we seek to clarify whether potent P2Y₁₂ inhibitors offer advantages in specific CCS subgroups, such as those with high thrombotic risk or complex PCI. The findings will address current guideline ambiguities and inform personalized antiplatelet strategies in this understudied population. 

## Review

Methodology 

Literature Search and Search Strategy 

This systematic review and meta-analysis followed the Preferred Reporting Items for Systematic Reviews and Meta-Analyses (PRISMA) guidelines. A comprehensive literature search was conducted using PubMed, Embase, Web of Science, and the Cochrane Central Register of Controlled Trials (CENTRAL) from inception to 5 May 2025. The search strategy included a combination of Medical Subject Headings (MeSH) terms and free-text keywords related to the intervention and population of interest. The primary search terms included: “ticagrelor,” “clopidogrel,” “percutaneous coronary intervention,” “chronic coronary syndrome,” “stable coronary artery disease,” and “antiplatelet therapy.” Boolean operators (AND/OR) were used to combine the terms appropriately. No restrictions were placed on language or publication date. Additionally, reference lists of relevant articles and systematic reviews were manually screened to identify any additional eligible studies. The search was performed by two authors independently and any disagreements between the two authors were resolved through consensus. 

Eligibility 

Studies were included if they enrolled adult patients (aged 18 years or older) with CCS undergoing PCI and compared ticagrelor with clopidogrel. Eligible studies were required to report at least one clinical outcome of interest, including all-cause mortality, major adverse cardiovascular events (MACE), myocardial infarction (MI), revascularization, stent thrombosis, minor bleeding, or major bleeding. We considered both randomized controlled trials (RCTs) and observational studies (prospective or retrospective cohorts) to provide a comprehensive assessment of the evidence. Studies were excluded if they focused exclusively on patients with acute coronary syndrome (ACS) without separately reporting outcomes for CCS patients, lacked a comparative control group, or did not report relevant outcomes. In addition, case reports, editorials, reviews, conference abstracts without full-text data, and duplicate publications were excluded from the final analysis. 

Study Selection 

Two independent reviewers screened titles and abstracts for relevance. Full texts of potentially eligible studies were retrieved and assessed for inclusion. Discrepancies between reviewers were resolved through discussion or consultation with a third reviewer. 

*Data Extraction* 

A standardized data extraction form was used. Two reviewers independently extracted data on the following variables: study ID (author, year, and country), study design, sample size, patient characteristics (mean age, sex, and comorbidities), intervention and comparator details (drug, dosage), follow-up duration, and outcomes of interest. Outcome data were extracted as the number of events and total participants per group for each outcome. Any disagreements were resolved by consensus or third-party adjudication. 

Risk of Bias Assessment 

The Cochrane Risk of Bias 2.0 tool was used for RCTs, while the Newcastle-Ottawa Scale (NOS) was used for observational studies to assess methodological quality. Each study was graded as low, moderate, or high risk of bias. 

Statistical Analysis 

All statistical analyses were conducted using Review Manager (RevMan) version 5.4.1. Dichotomous outcomes were pooled using the Mantel-Haenszel method and expressed as risk ratios (RRs) with 95% CIs. A random-effects model was applied considering potential clinical and methodological heterogeneity. Heterogeneity among studies was assessed using the I² statistic, with values of 25%, 50%, and 75% representing low, moderate, and high heterogeneity, respectively. Statistical significance was defined as a p-value <0.05. 

Results 

**Figure 1:**
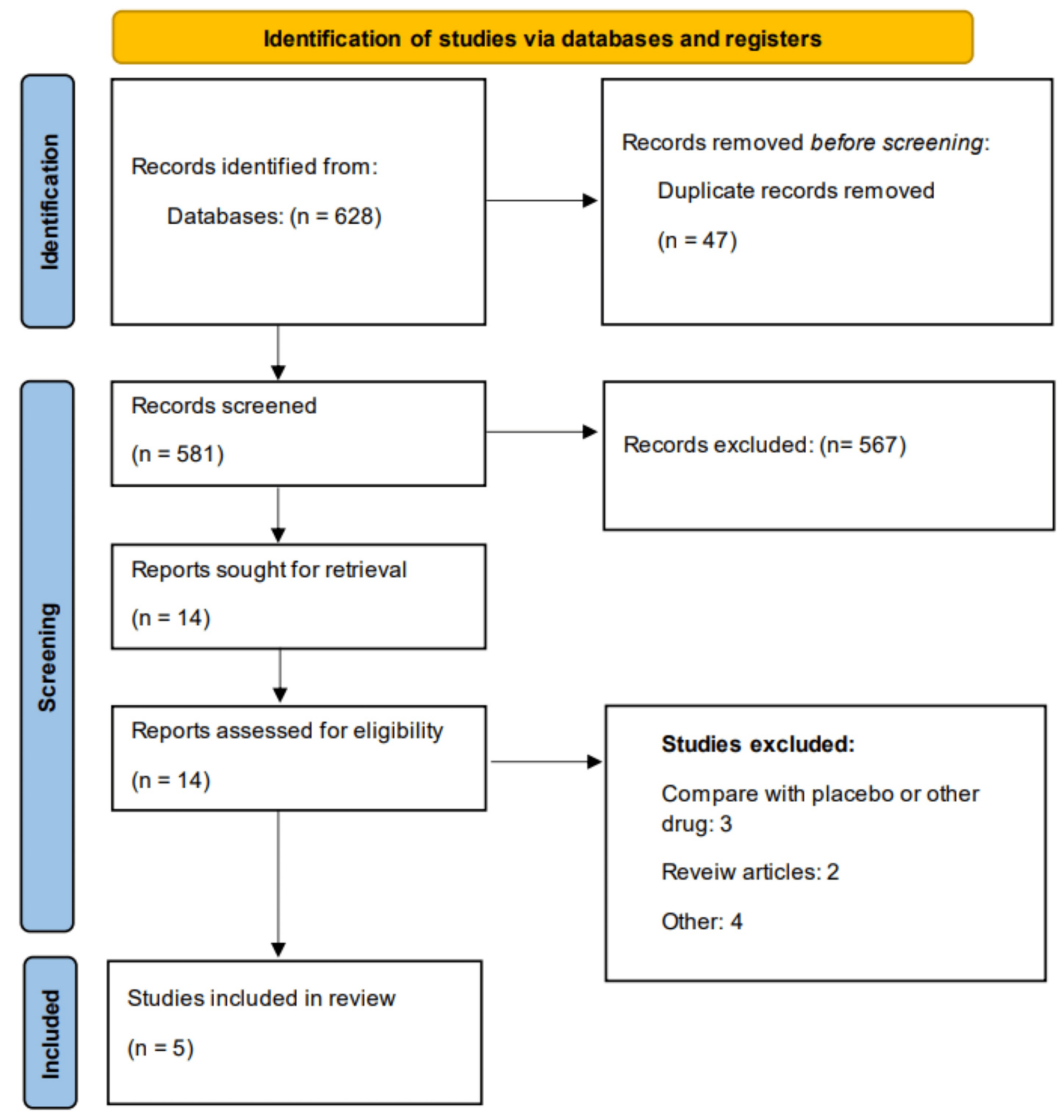
Study selection process (PRISMA flowchart) PRISMA, Preferred Reporting Items for Systematic Reviews and Meta-Analyses

Figure 1 presents the PRISMA flowchart of the study selection process. Through online database searching, we identified 628 studies. Initial screening of 581 records was done using abstracts and titles. Full text of 14 studies was obtained and detailed assessment was done. A total of five studies were included in this systematic review and meta-analysis, encompassing a combined population of patients with CCS undergoing PCI. Details of the included studies are presented in Table 1. Of these, two were RCTs, two were retrospective observational studies, and one was a prospective observational study. The studies were conducted across various regions, including Pakistan, China, the United States, and Europe, reflecting a diverse patient population. The total sample sizes for the ticagrelor and clopidogrel groups were 5,958 and 24,194, respectively. Follow-up durations ranged from one to 12 months. The mean age of participants varied between 57.1 and 66.2 years, and the majority of participants in each study were male. Reported comorbidities included diabetes and hypertension, with a notable proportion of patients affected in the studies that reported these variables. The ticagrelor loading dose was typically 180 mg, while clopidogrel dosing ranged from 300 to 600 mg. Table 2 presents the quality assessment of the included studies.

**Table 1:** Included study characteristics RCT: Randomized control trial; NR: Not reported

Study ID	Region	Study design	Groups	Dose	Sample size	Follow-up	Mean age (Years)	Males (n)	Diabetes (n)	Hypertension (n)
Koshy et al., 2023 [7]	United States	Retrospective observational	Ticagrelor	180 mg	1717	12 Months	NR	NR	NR	NR
			Clopidogrel	600 mg	8648					
Lattuca et al., 2024 [10]	Multicenter	RCT	Ticagrelor	180 mg	934	1 Month	66.2	1484	676	NR
			Clopidogrel	300 to 600 mg	932					
Li et al., 2021 [11]	China	Prospective observational	Ticagrelor	180 mg	1081	12 Months	58	1701	780	1414
			Clopidogrel	300 to 600 mg	1081					
Rauf et al., 2025 [9]	Pakistan	RCT	Ticagrelor	NR	150	3 Month	57.1	210	111	129
			Clopidogrel		150					
Xi et al., 2021 [12]	China	Retrospective observational	Ticagrelor	NR	2076	12 Months	59.2	12996	5091	9674
			Clopidogrel		13383					

**Table 2:** Quality assessment of included studies

Quality assessment of observational studies							
Study ID	Selection	Comparability	Outcome	Overall			
Koshy et al., 2023 [7]	4	1	3	Good			
Li et al., 2021 [11]	4	1	3	Good			
Xi et al., 2021 [12]	3	1	2	Good			
Quality assessment of observational studies							
Study ID	Randomization	Deviation from intended outcome	Missing outcome	Measurement of outcomes	Selection	Other bias	Overall
Lattuca et al., 2024 [10]	Low bias	High bias	Low bias	Low bias	Low bias	Unclear	Moderate
Rauf et al., 2025 [9]	Low bias	High bias	Low bias	Unclear	Low bias	Unclear	Moderate

*All-Cause Mortality* 

**Figure 2:**
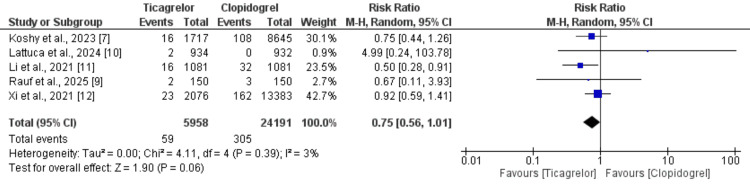
Comparison of risk of mortality

All five included studies assessed the risk of all-cause mortality between ticagrelor and clopidogrel in a total of 30,152 patients with CCS. The pooled analysis is presented in Figure 2 [7,9,10-12]. Overall, the all-cause mortality rate was lower in the ticagrelor group (0.99%) compared to the clopidogrel group (1.26%), with a pooled RR of 0.76 (95% CI: 0.57 to 1.01). Although the point estimate favored ticagrelor, the difference was not statistically significant, as the CI included the null value. Additionally, there was no significant heterogeneity among the studies (I²=3%). 

Major Adverse Cardiovascular Events

**Figure 3:**
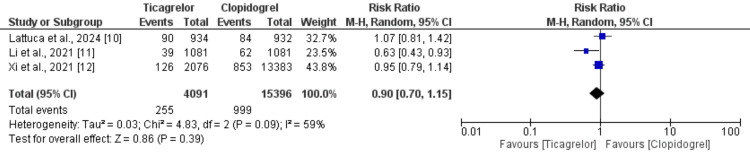
Comparison of risk of MACE MACE, major adverse cardiovascular events

Three of the five included studies reported on the incidence of MACE in patients with CCS, comprising a total of 19,487 participants. The pooled results, illustrated in Figure 3 [10-12], demonstrated no significant difference in the risk of MACE between the ticagrelor group (6.23%) and the clopidogrel group (6.48%), with a pooled RR of 0.90 (95% CI: 0.70 to 1.15). However, substantial heterogeneity was observed among the studies (I²=59%), suggesting variability in the study outcomes.

Myocardial Infarction

**Figure 4:**
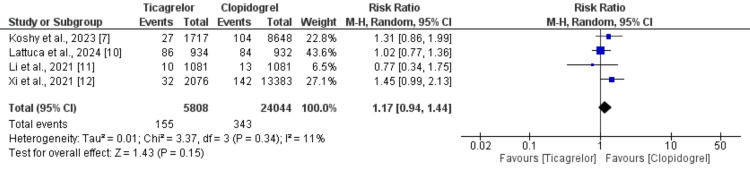
Comparison of risk of MI MI, myocardial infarction

Four of the five studies evaluated the risk of MI between patients receiving ticagrelor and those receiving clopidogrel, involving a total of 29,582 individuals with CCS. As shown in Figure 4 [7,10-12], the pooled analysis indicated that the incidence of MI was slightly higher in the ticagrelor group (2.67%) compared to the clopidogrel group (1.43%), with a RR of 1.17 (95% CI: 0.94 to 1.44). This difference was not statistically significant, and heterogeneity among the study findings was low (I²=11%). 

Revascularization 

**Figure 5:**
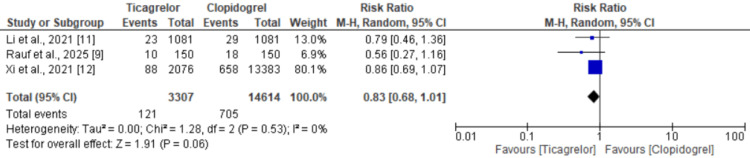
Comparison of risk of revascularization

All four studies that reported on revascularization outcomes in CCS patients comparing ticagrelor with clopidogrel included a total of 17,921 participants. Figure 5 [9,11,12] presents the pooled analysis, which revealed no significant difference in the rate of revascularization between the ticagrelor group (3.66%) and the clopidogrel group (4.82%). The pooled RR was 0.83 (95% CI: 0.68 to 1.01), favoring ticagrelor but not reaching statistical significance. No heterogeneity was detected among the included studies (I²=0%).

Safety Outcomes 

Safety outcomes including stent thrombosis, minor bleeding, and major bleeding were reported across the included studies and are summarized in Table 3. The risk of stent thrombosis was significantly lower in the ticagrelor group (13 events among 2,165 patients) compared to the clopidogrel group (26 events among 2,163 patients), with a pooled RR of 0.50 (95% CI: 0.26 to 0.97), and no heterogeneity observed (I²=0%). In contrast, the risk of minor bleeding was also not significantly different between ticagrelor (96 events among 3,307 patients) and clopidogrel group (165 events among 14,614 patients), with an RR of 1.66 (95% CI: 0.97 to 2.85), and substantial heterogeneity across studies (I²=72%). For major bleeding, the incidence was also higher with ticagrelor (50 events among 4,241 patients) compared to clopidogrel (105 events among 15,546 patients), although the difference was not statistically significant (RR: 1.28, 95% CI: 0.87 to 1.89), with no heterogeneity detected (I²=0%).

**Table 3:** Safety events analysis RR: risk ratio

Outcomes	Number of events in ticagrelor/total subjects)	Number of events in clopidogrel/total subjects	RR (95% CI)	I-Square
Stent thrombosis	13/2165	26/2163	0.50 (0.26 to 0.97)	0%
Minor bleeding	96/3307	165/14614	1.66 (0.96 to 2.85)	72%
Major bleeding	50/4241	105/15546	1.28(0.87 to 1.89)	0%

Discussion 

CCS refers to the long-term, stable phase of coronary artery disease and encompasses a spectrum of clinical presentations characterized by myocardial ischemia due to atherosclerotic plaque build-up and impaired coronary blood flow [13]. CCS includes patients with stable angina, silent myocardial ischemia, and those with a history of MI or coronary revascularization [14]. The diagnosis of CCS relies on a combination of clinical assessment, evaluation of symptoms (typically exertional chest discomfort), stress testing, imaging modalities to assess myocardial perfusion or coronary anatomy, and a thorough review of the patient’s cardiovascular history and risk factors [15]. 

This study aims to evaluate and contrast the clinical outcomes associated with two widely prescribed antiplatelet agents, ticagrelor and clopidogrel, in patients diagnosed with CCS who have undergone PCI. In our meta-analysis, ticagrelor was assessed as the treatment of interest, while clopidogrel served as the comparator. Despite both medications demonstrating significant therapeutic value, there is still ongoing debate regarding the superiority of one over the other. Through this systematic review, we conducted a detailed investigation to assess their respective efficacy and safety profiles. 

Our findings suggest that while ticagrelor was associated with a numerically lower risk of all-cause mortality and revascularization compared to clopidogrel, these differences did not reach statistical significance. Similarly, no significant difference was observed between the two drugs in reducing the incidence of MACE and MI, although the point estimates slightly favored ticagrelor in some outcomes. Importantly, ticagrelor significantly reduced the risk of stent thrombosis but was associated with a slightly higher incidence of minor bleeding but the difference was not statistically significant. In some of the outcomes, high heterogeneity was reported including MACE and minor bleeding. However, due to the limited number of studies included in this meta-analysis, we were unable to perform a subgroup analysis. 

Globally, ticagrelor has consistently outperformed clopidogrel in terms of MACE reduction, as revealed by meta-analyses [16] and investigations on stable coronary artery disease. The TALOS-AMI trial demonstrated the advantages of moving from ticagrelor to clopidogrel in high-risk individuals, balancing effectiveness and safety [17]. The present meta-analysis also supported the findings that MACE is lower in subjects receiving ticagrelor. However, only three studies compared this outcome. We need more studies to compare outcomes in CCS subjects. 

The present study reported a higher incidence of minor and major bleeding events in ticagrelor compared to clopidogrel, but the difference was statistically insignificant. Similarly, the trials including patients with ACS did not show ticagrelor is associated with an increased risk of bleeding events [18,19]. However, in patients with CCS, there is a lack of data. 

The 2021 guidelines from the American College of Cardiology (ACC), American Heart Association (AHA), and Society for Cardiovascular Angiography and Interventions (SCAI) on coronary revascularization highlight the limited availability of evidence supporting the use of ticagrelor and prasugrel in patients with CCS undergoing PCI [20]. On the other hand, the European Society of Cardiology (ESC) guidelines offer a Class IIb recommendation for using ticagrelor or prasugrel in elective patients with CCS who are at elevated risk of stent thrombosis or undergoing complex procedures such as left main or multivessel PCI, though this recommendation is based on limited evidence (Level of Evidence C) [21]. 

Study Limitations

This meta-analysis has several important limitations. First, only five studies were included, of which only two were RCTs; the remaining were observational in nature, which may introduce confounding and selection bias. Second, there was notable variability in the demographic characteristics of the study populations, particularly with respect to ethnicity and race, which may limit the generalizability of the findings. Third, two of the included studies were conducted in China, introducing a potential geographical bias that may not reflect outcomes in other regions or healthcare systems. Fourth, the duration of follow-up in two studies was limited to just one to three months, making it difficult to draw firm conclusions regarding the absence of long-term adverse outcomes, particularly over a 12-month period. Finally, because majority of the studies did not report data based on subgroups. Therefore, subgroup analyses for patients with important comorbidities such as chronic kidney disease, diabetes mellitus, malignancy, and other chronic health conditions could not be performed, limiting our ability to assess the consistency of the findings across clinically relevant subpopulations. These limitations should be taken into account when interpreting the results of this meta-analysis.

The findings of this meta-analysis have important implications for both clinical practice and future research. Clinically, while ticagrelor showed a potential advantage in reducing stent thrombosis, its benefits in terms of all-cause mortality, MACE, and MI were not statistically superior to clopidogrel, highlighting the need for individualized treatment decisions. Given the higher, though not significant, bleeding risk with ticagrelor, careful patient selection is essential. From a research perspective, larger, high-quality RCTs focusing specifically on CCS populations are needed to confirm these findings and explore subgroup effects based on comorbidities, risk profiles, and procedural complexity to guide more precise antiplatelet therapy. 

## Conclusions

This systematic review and meta-analysis evaluated the comparative efficacy and safety of ticagrelor versus clopidogrel in patients with CCS undergoing PCI. While our findings did not demonstrate statistically significant differences in all-cause mortality, MACE, MI, or revascularization between the two antiplatelet agents, ticagrelor showed a statistically significant reduction in stent thrombosis, though this finding should be interpreted cautiously given the low absolute number of events across contributing studies. The numerically higher bleeding rates with ticagrelor, while not reaching statistical significance, represent a clinically relevant consideration that may influence therapeutic decisions, particularly in patients with elevated baseline bleeding risk. These findings are based on a limited number of predominantly observational studies with significant heterogeneity, limiting definitive conclusions. These results highlight the importance of individualized antiplatelet therapy in CCS patients, carefully weighing baseline bleeding risk (HAS-BLED score), procedural complexity (multivessel intervention and bifurcation stenting), and comorbid conditions (diabetes, prior MI, and chronic kidney disease) when selecting optimal strategies.

The limited number of high-quality randomized trials specifically addressing CCS populations represents a significant knowledge gap. Large-scale, adequately powered RCTs are urgently needed to confirm these preliminary signals and establish definitive risk-benefit profiles. Future research should prioritize identifying specific CCS subgroups that may derive greater benefit from potent P2Y₁₂ inhibition while minimizing bleeding risk. Until robust randomized evidence emerges, clinicians should integrate these hypothesis-generating findings with individual patient risk profiles to optimize antiplatelet selection in CCS management.
